# Effect of Different Luting Cements on Fracture Resistance in Endodontically Treated Teeth

**Published:** 2008-10-01

**Authors:** Narmin Mohammadi, Amir Ahmad Ajami, Soodabeh Kimyai, Mojdeh Rezaei Aval

**Affiliations:** 1 *Department of Operative Dentistry, Dental School, Tabriz University of Medical Sciences, Tabriz, Iran*; 2 *Department of Operative Dentistry, Dental School, Babol University of Medical Sciences, Babol, Iran*

**Keywords:** Composite Resin, Endodontically Treated Teeth, Fracture Resistance, Luting Cement, Post

## Abstract

**INTRODUCTION:** The aim of the present study was to evaluate the effect of three types of luting cements used for post cementation on the fracture resistance of endodontically treated maxillary premolars, restored with resin composite.

**MATERIALS AND METHODS:** One hundred intact single-rooted human maxillary premolars were randomly divided into 5 groups of 20 each. In groups 2-5, post spaces were prepared after root canal treatment and clinical crown reduction up to 1.5 mm above the CEJ. Teeth were divided in groups as follows: Group 1: intact teeth, Group 2: active prefabricated metallic posts (PMP), Group 3: PMP cemented with zinc phosphate luting cement, Group 4: PMP cemented with glass ionomer luting cement and Group 5: PMP cemented with resin luting cement. In groups 2-5 the teeth were restored with resin composite. Following thermocycling, the palatal cusp of each specimen was loaded to compression at an angle of 150˚ to its longitudinal axis at a strain rate of 2 mm/min until fracture occurred. Data were analyzed using one-way ANOVA and a post hoc Tukey test. Chi-square test was used for comparison of failure mode.

**RESULTS:** There were significant differences in fracture resistance between the test groups (P<0.001). The differences between group 2 with groups 1, 4 and 5 were statistically significant (P<0.05); whereas there were no significant differences in fracture resistance between the two other groups (P>0.05). Furthermore, there were no significant differences in the mode of failure between the 5 groups (P>0.05).

**CONCLUSION:** Zinc phosphate, glass ionomer and resin luting cements showed similar behaviors and achieved fracture resistance comparable to intact teeth. However, the use of active post (without cement) adversely affected the fracture resistance of root canal treated teeth.

## INTRODUCTION

Fracture of endodontically treated premolars is a common problem encountered in the clinical setting. Numerous reports have documented a high incidence of fracture for endodontically treated maxillary premolars (1-3). Root canal treated posterior teeth are subjected to greater loading than anterior teeth because of their closer proximity to the transverse horizontal axis. This, combined with their morphologic characteristics (having cusps that can be wedged apart), makes them more susceptible to fracture (4). Moreover, they often have lost a large proportion of coronal tooth structure as a result of dental caries, previous restorations and/or endodontic treatment (4,5). 

Cast post-core restorations have been used to strengthen endodontically treated teeth (6). Nevertheless, studies have shown that dislodgment and root fracture still occur (6,7). Pre-fabricated post systems have become popular because of their satisfactory results. In addition, they save chair time and can reduce the cost (8). However, selection of a cementing medium still continues to be a clinical challenge. It has been reported that the type of luting cement can influence the fracture resistance of root canal treated teeth (9).

Different luting agents are used for post cementation. Zinc phosphate cement is one of the most frequently used cements in dentistry because of its well-known clinical characteristics and long history of effectiveness (10), though some of its properties are inferior to those of some recently developed cements (11). Although it lacks adhesiveness to tooth structure and has no anticariogenic properties (12), zinc phosphate cement can adhere to post and root irregularities by mechanical retention (13). In comparison, glass ionomer cement adheres to tooth structure by chemical bonding. The chemical reaction is ionic and occurs between the carboxyl ions of polyacrylic acid and the calcium in tooth structure (14). The clinical advantages include ease of application, chemical adhesion to dental tissue and fluoride release (15). Resin cements adhere to tooth structure by the hybrid layer, an intermediate zone created by impregnation, diffusion, and monomer polymerization into the dentin which has previously been etched by acid conditioners (13). Thus, the resin cement promotes bonding to dentin in a different manner when compared with other cements, and the literature demonstrates the superiority of these luting agents in tensile bond strength (13,16).

Little is known about the effect of different luting agents on fracture resistance of root canal treated teeth restored with resin composite. Therefore, the purpose of the present study was to evaluate the effect of zinc phosphate, glass ionomer and resin luting cements used for post-cementation on fracture resistance of endodontically treated premolars.

## MATERIALS AND METHODS

The compositions of the materials used in the present study are shown in Table 1. One hundred intact single-rooted human maxillary premolars with similar root sizes were selected from a supply of maxillary premolars extracted for orthodontic reasons within a two-month period. The teeth were free of caries, previous restorations and pre-existing fractures or cracks when surveyed under light microscope (Olympus, Model CH30RF200, Olympus Optical Co., LTD, Japan). The teeth were stored in 0.5% Chloramine T Trihydrate at 4˚C for control infection. A hand scaling instrument was used for surface debridement of the teeth, followed by cleaning with a rubber cup and slurry of Pumice (Pumice Preppies, Whip Mix Europe GmbH, Dortmund, Germany).

Using a simple random sampling method, the teeth were divided into 5 groups of 20 each. Group 1 consisted of intact teeth. In groups 2 to 5 the clinical crowns of the teeth were removed 1.5 mm coronal to the CEJ with a diamond bur (SS White Burs, Inc. Lakewood, NJ, USA) in a high-speed handpiece (Bien-Air SA, Länggasse 60, Case postale 8, CH-2500 Bienne 6, Switzerland) under constant water spray, followed by standard access cavity for endodontic treatment using a coarse tapered flat-end diamond bur (TF-13C, Mani, Japan). During tooth preparation each tooth was wrapped in water moistened gauze. A new bur was used after every 10 preparations. Each canal was prepared up to 1 mm from the radiographic apex. The root canal of each tooth was instrumented using passive step-back technique to a #35 K-files (MANI, Inc., Tochigi, Japan) at the apical constriction. The obturation was performed with gutta-percha and AH26 root canal sealer (Dentsply DeTrey GmbH, Konstanz, Germany), using lateral condensation technique. Then long #2 threaded tapered prefabricated posts (Dental Gold Plated Screw Posts, Nordin SA, Chailly-Montreux, Switzerland) were selected. Post spaces were prepared with Peeso reamers #1, 2, and 3 (LARGO, Dentsply Maillefer, Ballaigues, Switzerland) up to 8 mm apical from the CEJ. The post spaces were rinsed with distilled water for 15 seconds and dried with paper points (Aria Dent, Tehran, Iran). The cements were then mixed according to manufacturers' instructions at room temperature (23 ± 1˚C) and placed in root canals by a lentulo spiral (Dentsply Maillefer, Ballaigues, Switzerland) using a low speed contra-angle handpiece (Bien-Air, Switzerland). The posts were placed in the canals after being coated with cement.

The specimens were prepared as follows:

Group 1: intact teeth,

Group 2: prefabricated metallic posts placed actively in the canal. In this group, posts were screwed into the canal with a screwdriver, 

Group 3: prefabricated metallic posts cemented with zinc phosphate luting cement (Harvard Dental GmbH, Berlin, Germany), Group 4: prefabricated metallic posts cemented with glass ionomer luting cement (Fuji I, GC, Tokyo, Japan), and Group 5: prefabricated metallic posts cemented with dual-cured resin cement (Rely X ARC, 3M ESPE, St. Paul, MN, USA).

**Table 1:** The materials used in this study

Material	Compositions	Batch number
Adper Single Bond	Light-cured adhesive system containing 2-HEMA, Bis-GMA, Amine di-methacrylate, Polyalkanoic acid, Itanoic acid , Ethanol, Water	6KR
Filtek Z250	Light-cured composite resin, radiopaque , containing Zirconia/ silica filler (60% wt – particle range 0.01- 3.5 µm), Bis –GMA, UDMA, Bis-EMA, TEG DMA	7XT
Zinc phosphate cement	Powder/liquid; powder: ZnO, MgO, SiO_2_, Misc, Ba_2_SO_4_,CaO; liquid: Zn, Al, H_3_PO_4_, H_2_O	Powder: 1940605 Liquid: 1900606
Glass ionomer cement	Powder/liquid, self-curing, powder: Flouroaluminosilicate glass, Potassium persulfate and acid ascorbic catalyst; liquid: Water solution of acid polycarboxilic modified with 2-HEMA and Tartaric acid	0410061
Rely-X ARC	Dual-curing cement, radiopaque, Bisphenol-A-diglycidyether dimethacrylate (BisGMA), Triethylen glycol dimethacrylate (TEGDMA), Zirconia/silica filler, Dimethacrylate monomers	20050608

Excess cement was removed and a bevel of 45˚ (with 0.5 mm width) was placed at the periphery of the remaining crown. The coronal structure was etched with 35% phosphoric acid gel (Scotch Bond Etchant, 3M ESPE, St. Paul, MN, USA). After rinsing and removing the excess water, a bonding agent was applied according to manufacturer's instructions (Adper Single Bond, 3M ESPE, St. Paul, MN, USA) and cured for 10 second using an Astralis 7 light-curing unit (Ivoclar Vivadent Inc., Amherst, NY, USA) adjusted to 400 m W/cm^2^. Then a stainless steel matrix band (Tofflemire Matrix Band/Henry Schein, Inc, Melville, NY, USA) with a retainer was placed around the teeth and resin composite (Filtek Z250, 3M ESPE, St. Paul, MN, USA) was built up in two 1.5 mm-thick layers on the bonded surface. Each layer was light-cured for 40 seconds with the Pulse Program of the light-curing unit, from occlusal aspect. In the Pulse Program, initial light curing used low intensity light (150 mW/cm^2^) for 15 seconds, followed by a gradual increase in intensity (up to 700 mW/cm^2^) until the 40 seconds of exposure time is completed. In order to form a similar cuspal incline in all the specimens, one-third of the occlusal portion of a prefabricated transparent (polyvinyl chlorite) premolar crown** (**TDV Dental, Pomerode-SC, Brazil**),** which had been sectioned previously, was used. Resin composite was placed inside the restorative crown as cementing medium. Then the restorative crown was placed over the previous composite layers to restore the occlusal part of the specimens. Resin composite was cured with the Pulse Program of the light-curing unit before removing the transparent crown. Post-curing was carried out on each side of the teeth for 40 seconds at an intensity of 700 mW/cm^2^. After finishing using finishing diamond burs (D&Z, Berlin, Germany) and polishing with polishing disks (Sof-LexTM, 3M ESPE, St. Paul, MN, USA), the specimens were stored in distilled water at 37˚C for one week, and thermocycled at 5˚C±5˚C / 55˚C±5˚C (500 times) with a dwell time of 30 seconds and 10 seconds for specimen transfer. After reconstruction, all the specimens were embedded in cold-cured acrylic resin up to 1.5 mm apical to the CEJ.

Fracture resistance was evaluated in a universal testing machine (Model H5K-S, Hounsfield Test Equipment, Raydon, England) using loads at 150˚ to the root long axis (30˚ to the horizontal plane) and compression load was applied on the buccal incline of the palatal cusps, at a strain rate of 2 mm/min until fracture occurred. Fractures were divided into two groups based on the their extent: A) fractures stopping at ≤ 1 mm below the embedding resin surface (favorable fractures) and B) fractures terminating at ≥1 mm apically, below the embedding surface (unfavorable fractures).

Data were analyzed using one-way ANOVA (P<0.05) and a post hoc Tukey test. Chi-square test was used for the comparison of the mode of failure.

**Table 2:** Statistical indexes of fracture resistance for each test Group

Group	N	Mean (SD)	Min	Max
1	20	829.25 (265.05)	418	1422
2	20	409.25 (185.26)	265	778
3	20	584.65 (186.23)	354	860
4	20	825.15 (193.42)	549	1048
5	20	842.65 (196.27)	578	1134

## RESULTS

The means of fracture resistance in all groups are listed in Table 2. According to one-way ANOVA analysis, there were significant differences in the mean fracture resistance among all groups (f_ (4,95)_ = 5.92, P<0.001). The maximum and minimum values were related to group 5 and group 2, respectively. The post hoc Tukey test revealed that the differences between groups 1 and 2 (P=0.003), groups 2 and 4 (P=0.003), and groups 2 and 5 (P=0.002) were statistically significant. However, there were no significant differences between other groups.

Among patterns of fracture, the maximum number of favorable fractures was observed in group 1 and the minimum number was observed in group 2 (Table 3). According to chi-square test, there were no significant differences in the fracture mode of groups (χ^2 ^=5.08, df = 4, P= 0.281) (Table 3).

## DISCUSSION

In this study, maxillary premolars were used since these teeth have been reported to show a high incidence of fracture in the clinical setting (6).

According to the results of the present study there were no statistically significant differences in the fracture resistance of three groups with cemented posts (groups 3, 4 and 5) and the type of cements was not a determining factor in fracture resistance of endodontically treated premolars. Our results confirm findings reported by Mezzomo *et al.* that fracture resistance of cast post and cores with zinc phosphate and resin cement was not significantly different (10).

**Table 3:** Fracture patterns in test groups

***Mode of failure***		
Group	Favorable	Unfavorable
1	13 (65%)	7 (35%)
2	6 (30%)	14 (70%)
3	10 (50%)	10 (50%)
4	9 (45%)	11 (55%)
5	9 (45%)	11 (55%)

In a study carried out by Yamada *et al*., with the aim of comparing fracture resistance of endodontically treated maxillary premolars restored with metallic inlays and onlays, it was demonstrated that fracture resistance of the teeth in which zinc phosphate had been used was significantly lower than that with the use of resin cement (6). The difference in results could reflect the variation in type of restorations, preparation, design, experimental materials, and thermo-cycling.

In the current study, there were no significant differences between three cemented post groups and intact teeth (group 1). This lack of difference might be attributed to the reinforcing effect of bonded resin composite, used to restore endodontically treated teeth. Several studies have reported that fracture resistance of premolars restored using resin composite is comparable to that of intact premolars (17,18). Furthermore, it might be due to high filler content, better mechanical properties and the modulus of elasticity of hybrid resin composite (Filtek Z250), which is comparable to dentin (9,19). It has been reported that the physical resistance of teeth is significantly improved when they are restored with materials that have low modulus of elasticity rather than with hard materials because the loads are better absorbed and the restoration works as a homogeneous block (10).

When the fracture resistance in group 2 was compared to that in groups 1, 4, and 5, there were significant differences but the difference between groups 2 and 3 was not significant. Posts that are placed actively cannot distribute stresses in an uniform pattern and majority of stresses appear at apical end of the posts (20,21). Moreover, it has been pointed out that by using cements which were bonded to root canal walls, a buffer zone was provided that contributes to uniform distribution of stress transmission from the posts to canal walls via the sandwiched cement (8). In group 2, in which the posts were placed actively without any cement, and in group 3, in which zinc phosphate (non-adhesive luting cement) was used, the above-mentioned buffer zone was not observed. Therefore, fracture resistance was lower than the other groups (1, 4, and 5).

Differences between groups 1 and 2 were significant, which might be attributed to the fact that sound tooth structure is a complex system with reinforcing components that do not easily allow progression of fracture (22).

Even though minimum fracture resistance in all the groups except group 2was more than the normal range of biting force for maxillary premolars [100-300 N] (23), fracture resistance was recorded using a destructive test that may not always simulate *in vivo* conditions. In this method the static load was applied until fracture occurs whereas the forces in the oral cavity are dynamic (24,25). Furthermore, fatigue failure may occur in the mouth (26). Consequently the results should be interpreted cautiously and long term clinical studies are warranted. In addition, other studies should simulate periodontal ligament to further replicate clinical situations.

Regarding fracture mode, though there were no significant differences between groups, the most favorable fractures were observed in group 1, which might be attributed to the preservation of tooth structure and the uniform distribution of loads. The most unfavorable fractures were detected in group 2, one could attribute it to the stresses generated in the root canal following insertion of active posts. Active posts do not distribute stresses evenly and can produce high stress concentrations during insertion and loading while the cement layer results in a more even stress distribution to the root with less stress concentration (4,27).

## CONCLUSION

Considering the limitations of this study:

1. Zinc phosphate, glass ionomer, and resin luting cements used for post cementation did not reveal any significant differences in fracture resistance of endodontically treated teeth.

2. There were not any significant differences in the fracture resistances of intact teeth and three cemented post groups.

3. Active posts significantly decreased the fracture resistance of endodontically treated teeth.

4. Intact teeth had the most favorable fractures while teeth restored with active posts demonstrated the most unfavorable fractures.
